# Measurement innovation for sensitive behaviours: applying direct and social network-based estimation approaches to intimate partner violence in Burkina Faso and the Democratic Republic of the Congo using cross-sectional data

**DOI:** 10.1136/bmjopen-2025-114411

**Published:** 2026-05-26

**Authors:** Haley L Thomas, Suzanne O Bell, Fiacre Bazié, Georges Guiella, Dynah M Kayembe, Pierre Z Akilimali, Michele R Decker, Shannon N Wood

**Affiliations:** 1 Department of Population, Family and Reproductive Health, Johns Hopkins Bloomberg School of Public Health, Baltimore, Maryland, USA; 2 Institut Supérieur des Sciences de la Population, Joseph Ki-Zerbo University, Ouagadougou, Centre Region, Burkina Faso; 3 Kinshasa School of Public Health, University of Kinshasa, Kinshasa, Congo (the Democratic Republic)

**Keywords:** Gender-Based Violence, Surveys and Questionnaires, Africa South of the Sahara

## Abstract

**Abstract:**

**Objectives:**

To explore the feasibility of the confidante methodology to measure past-year intimate partner violence (IPV) experiences in Burkina Faso and the Democratic Republic of the Congo (DRC) through (1) comparison of direct assessment with indirect estimation via the confidante method and (2) assessment of the performance of each confidante method assumption.

**Design:**

Cross-sectional study with nationally and subnationally representative data collected from December 2020 to March 2021 in Burkina Faso (national) and from December 2021 to April 2022 in Kinshasa and Kongo Central, DRC (subnational).

**Setting:**

Burkina Faso; Kinshasa, DRC; Kongo Central, DRC.

**Participants:**

Partnered women (married or cohabiting) aged 15–49 in Burkina Faso (N=3047), Kinshasa, DRC (N=702) and Kongo Central, DRC (N=688) and their partnered confidantes aged 15–49 (N=2064 in Burkina Faso, N=304 in Kinshasa, DRC, N=393 women in Kongo Central, DRC).

**Primary and secondary outcome measures:**

Past-year IPV (emotional, physical, sexual, any) comparing differences in prevalence between the direct respondent sample and the indirect confidante sample, adjusting for confidante method assumptions.

**Results:**

The confidante method produced comparable IPV estimates to respondents’ direct reports across sites (35.3% respondent vs 36.1% confidante in Kinshasa, DRC; 29.7% respondent vs 39.0% confidante in Kongo Central, DRC; 25.7% respondent vs 26.0% confidante in Burkina Faso, differences not statistically significant). Of note, there were differences in IPV estimates between respondents and confidantes by IPV subtype, with physical IPV consistently lower among respondents across sites and sexual IPV lower among confidantes in Kinshasa, DRC and Burkina Faso, though generally not statistically significant.

**Conclusions:**

The confidante methodology did not afford advantages over standard, direct assessment for IPV. Overall, findings indicate the reliability of population-based surveys with direct IPV questions when implemented under recommended ethical guidelines, though direct reports are likely undercounts.

**STRENGTHS AND LIMITATIONS OF THIS STUDY**

- This study is the first application of the confidante methodology for intimate partner violence (IPV) estimation.
- We collected and analysed socio-demographic data for both respondents and their confidantes to thoroughly apply the methodology and test underlying assumptions.
- We did not have information about the extent of transmission of IPV experiences between confidantes and respondents in Burkina Faso, though these data were available for the Democratic Republic of the Congo sites.
- Across sites, we were unable to distinguish the type of confidante and recognise that sharing of experiences may differ specifically for family versus friends.
- We were unable to determine differential transmission of IPV by IPV subtype, though hypothesise that more sensitive behaviours (eg, sexual IPV) may have been less likely to be shared.

## Introduction

 Intimate partner violence (IPV) refers to the physical, sexual or psychological violence perpetrated by a current or former partner or spouse.^1^ IPV is a human rights violation with far reaching implications given its high global prevalence and association with adverse health consequences.^1 2^ While IPV is prevalent across all social groups, it disproportionately impacts women, with one in three self-reporting experiences of IPV over the course of her lifetime.^1^ IPV is associated with poor mental health and physical injury, as well as a range of negative sexual and reproductive health outcomes such as HIV/sexually transmitted infection acquisition, unintended pregnancy and induced and spontaneous abortion.^1 3^ Accurately estimating the prevalence of IPV and identifying the subpopulations of women most at risk is pertinent to adequately designing and implementing violence prevention and response programmes, and assessing if, when, and for whom those programmes work.

Currently, IPV prevalence is most often estimated from population-based surveys via validated direct, behavioural assessment about respondents’ own IPV experiences.^1^ Population-based surveys are a critical data source for estimating IPV prevalence for generalisability; the well-documented underreporting of IPV to law enforcement, health centres and other formal sources introduces significant bias and undercounting in police, medical and service utilisation records, as many survivors will never seek help or disclose these experiences.^4^ Population-based survey methods offer significant strengths in unbiased sampling; however, there remains a risk of under-reporting due to the sensitive nature of IPV.^1^

Alternative measurement strategies, such as indirect measurement, can mitigate misreporting for sensitive topics like IPV. Indirect measurement methods in surveys allow respondents to answer sensitive questions without having to disclose their personal experiences directly, providing an opportunity to evaluate under-reporting from direct questions.^5^ Thus far, few quantitative indirect measurement methods have been used to estimate IPV, limited to the list experiment (also known as the item count technique) and the neighbourhood method. With the list experiment method, respondents are randomised to either a treatment or control group; the control group is read a list of non-sensitive items while the treatment group is read the same items, plus the sensitive item of interest (in this case, IPV). Respondents then report how many of the items they have experienced (without disclosing *which* items), and the difference in the total items between the treatment and control group is estimated, resulting in the prevalence of the sensitive item. Findings from studies using the list experiment to measure IPV are mixed—while some studies have found no significant differences in IPV prevalence when comparing the list experiment to direct reports,^6 7^ others have found that the list experiment yields higher IPV estimates^8 9^ or that estimates vary by IPV subtype (ie, physical IPV, sexual IPV).^10 11^ Additionally, this approach has limitations as it is statistically inefficient, and researchers are not able to collect additional details about the IPV experience.

The neighbourhood method is an indirect measurement technique developed by Stark and colleagues for measuring gender-based violence in complex humanitarian settings, where war and displacement present logistical and methodological challenges to obtaining quick and reliable prevalence data for programmatic purposes.^12^ This method builds on the sisterhood method (developed for maternal mortality estimation)^13^ by expanding the scope of who respondents report on, operating on the principle that individuals often have knowledge about the violence experiences of those in their immediate proximity or within their social network.^12^ With the neighbourhood method, selected women are interviewed about violence across multiple populations including their own personal experiences, those of other women in their household, those of women in neighbouring households, and in some cases, those of their sisters. Since its development, the neighbourhood method has been employed to estimate IPV across diverse humanitarian contexts—including conflict zones, refugee camps and post-conflict settings—with variation between direct and indirect prevalence estimates across IPV types and the population being reported on.^12 14 15^

Another indirect method that relies on third-party reporting of the sensitive behaviours of individuals in survey respondents’ social networks is the best friend or confidante method, sometimes referred to as the anonymous third-party reporting method.^16^,^30^ This method asks respondents if they have a person in their social network, such as a confidante, with whom they share personal information. If a person is identified, questions can then capture individual-level data of the confidante, such as socio-demographic characteristics, in addition to information on the sensitive behaviour of interest. This results in a detailed confidante sample alongside the respondent sample, adding value and precision to the indirect measurement of sensitive behaviours by enabling researchers to identify who is most at risk of the behaviour and related outcomes among both samples, which can then be compared. The confidante method has three broad assumptions: (1) characteristics of the confidante sample resemble the characteristics of the respondent sample, providing a representative, surrogate sample of the population of interest; (2) respondents know about their confidante’s experiences of interest, that is, there is no transmission bias between respondents and their confidantes; (3) reporting on a confidante’s experience, as opposed to oneself, reduces social desirability bias.^31^,^34^ When these assumptions are met, the resulting estimates are unbiased. This approach has been used to measure abortion, which is highly under-reported, with varying degrees of success,^1617 19 21^,^23 25 26^ as well as sexual activity and contraceptive use among adolescent girls,^30^ and could be extended to other sensitive behaviours such as IPV.

Previous research has highlighted that many women hesitate to share their IPV experiences with others out of fear of negative repercussions such as shame, social isolation or ostracisation and blame from themselves or others.^1^ When women do disclose their IPV experiences, they most frequently turn to informal social supports such as close friends or family.^35^,^42^ As such, social network-based indirect methods, including the confidante method, could be used to leverage the sharing of IPV experiences between respondents and their close friends or family to more accurately estimate IPV within populations; however, this methodology has not yet been assessed for IPV estimation. The aim of this study is to explore the feasibility of the confidante methodology to measure past-year IPV experiences in Burkina Faso and the Democratic Republic of the Congo (DRC) through (1) comparison of direct assessment with indirect report via the confidante method, and (2) assessment of the performance of each confidante method assumption. We describe the methods used for testing each assumption associated with the confidante methodology, based on established protocols from previous confidante studies and report results by each assumption.

## Methods

### Data

Data for this study come from Performance Monitoring for Action (PMA), a research platform that administers annual surveys at the household, woman and facility level in eight countries in sub-Saharan Africa and Asia. PMA uses a multistage stratified cluster sampling design with probability proportional to size selection of geographical clusters to obtain nationally or subnationally representative samples of households and reproductive-aged women. Trained interviewers administer surveys in-person using mobile phones with the Open Data Kit (ODK) software. Additional methodology details can be found at pmadata.org.

Analyses use national or subnational cross-sectional data from women collected in Burkina Faso (national; 19 December 2020 to 8 March 2021; response rate 93.5%), Kinshasa, DRC (subnational; 3 December 2021 to 25 April 2022; response rate 94.0%) and Kongo Central, DRC (subnational; 17 December 2021 to 25 April 2022; response rate 97.9%).^43 44^ We selected these sites for the present analyses as the corresponding surveys included a gender-based violence (GBV) module with embedded IPV questions for both the respondent and her confidante. All women in selected households aged 15–49 were eligible for the women’s survey; however, only one woman per household was eligible for the GBV module, per ethical guidelines. If there was more than one eligible woman per household, one was randomly selected. Among those selected for the GBV module, only those married or cohabiting (referred to as ‘partnered’ henceforth) were eligible for the IPV questions.

### Measures

To identify the confidante sample, respondents were asked if they had a confidante who was a woman between the ages of 15 and 49—consistent with the survey’s target population—who lived in the country and with whom they share very personal information. If the respondent had a confidante who met the definition criteria, interviewers collected information on the confidante’s age, education level, relationship status, parity, and residence from the respondent. If the respondent reported that her confidante was partnered, interviewers then asked about their confidante’s experiences with IPV in the last 12 months. Past-year IPV was measured via an abbreviated version of the Revised Conflict and Tactics Scale^45^ and assessed via affirmative response to any of the following items: In the last 12 months has her husband/partner: (1) insulted her, yelled at her, screamed or made humiliating remarks; (2) slapped, hit or physically hurt her; (3) threatened her with a weapon or attempted to strangle or kill her; (4) pressured or insisted on having sex when she did not want to (without physical force); (5) physically forced her to have sex when she did not want to. The subsequent section included direct questions about the respondent’s own experiences of past-year IPV, perpetrated by a husband or partner, using the same five items used to capture IPV experiences of the confidante (modifying language to ‘you’ instead of ‘her’). Respondents who had a confidante and experienced IPV were then asked if their confidante knew about their IPV, and if so, if they told their confidante about their past-year IPV experience directly (DRC sites only).

Non-mutually exclusive items for types of IPV were also created from individual items for both respondent and confidante data: emotional IPV (item 1), physical IPV (items 2–3), sexual IPV (items 4–5) or contact IPV, including sexual or physical IPV (items 2–5). Respondents missing self-reported IPV data or IPV data for their confidante were excluded from analyses (<1% across sites).

We examined socio-demographic characteristics for the confidantes and respondents, including age (15–29, 30–39, 40–49 in Kinshasa and Kongo Central; 15–19, 20–29, 30–39, 40–49 in Burkina Faso), education (none/primary, secondary, higher in Kinshasa and Kongo Central; none, primary, secondary or higher in Burkina Faso), residence (urban/rural) and parity (0, 1–2, 3+).

### Statistical analysis

Below we describe the methods used for testing each assumption associated with the confidante methodology, based on established protocols from previous confidante studies, and describe the approaches used to adjust for violations of assumptions, if detected.

### Assumption 1: confidante sample representativeness

To use the confidante sample to estimate population-level IPV prevalence, the confidantes must be representative of reproductive-aged women in the study geography. As all respondents did not report having a partnered confidante, these ‘missing’ confidantes could contribute to selection bias in the confidante sample. Additionally, the confidante sample may be less representative if respondents have confidantes who are systematically different from themselves. Since the respondent sample is designed to be representative of reproductive-aged women, we compared the distributions of socio-demographic characteristics between the respondent sample and the confidante sample using design-based F-tests. Given observed violations of this assumption, we created an adjusted confidante sample that included confidantes for those respondents who reported no partnered confidantes (ie, ‘missing’ confidantes). These ‘missing’ confidantes took on the socio-demographic characteristics of their corresponding respondents, a design choice rooted in the sociological concept of homophily—the principle that ‘birds of a feather flock together’—reflecting the tendency for social networks to exhibit similarity across demographic, behavioural and personal attributes.^34^ We then predicted the probability that these ‘missing’ confidantes would have experienced past-year IPV by fitting separate logistic regression models for each IPV type, regressing the respondents’ socio-demographic characteristics, including age, education, wealth tertile, urban/rural residence and parity, on the corresponding observed confidante IPV data, and imputing the resulting predicted probabilities (ranging from 0 to 1) for these confidantes. Additionally, we generated and applied post-stratification weights to the confidante sample to try and replicate the distribution of characteristics of the respondent sample.

### Assumption 2: transmission of IPV experiences

To assess if IPV experience information is fully transmitted from confidantes to respondents, we first evaluated whether respondents had confidantes, which is a pre-condition to sharing. We assessed differences in the distributions of socio-demographic characteristics between respondents who reported no confidantes and those with at least one confidante using design-based F-tests. We then evaluated whether respondents who reported any IPV told their confidante about it. In line with previous studies employing this method, we assumed that the respondent sharing mirrored the confidante sharing in the reverse direction. To adjust for transmission bias between confidantes and respondents, we generated a transmission bias adjustment factor or the inverse probability of respondents sharing their own reported IPV experiences with their confidante. This adjustment factor was then multiplied by the prevalence of IPV in the adjusted confidante sample. Adjustment was only possible in Kinshasa and Kongo Central based on this inclusion of survey items related to sharing experiences. Adjustment was also limited to any IPV overall rather than specific IPV subtypes, because respondents who reported any IPV were only asked about sharing of their experience(s) with confidantes in general terms, not about whether they shared each specific type of IPV experience.

### Assumption 3: reduced social desirability bias

To assess whether reporting on a confidante’s sensitive experience with IPV reduces social desirability bias, we first calculated the prevalence of past-year IPV for each sample (respondent, unadjusted confidante, adjusted confidante). For the adjusted confidante sample, prevalence was estimated by taking the mean across all confidante IPV values, combining binary outcomes (0 or 1) for those with an observed confidante and imputed predicted probabilities (0–1) for ‘missing’ confidantes. We then tested whether the prevalence of past-year IPV among the adjusted confidante samples was statistically different from the prevalence of past-year IPV among the respondent samples using z-tests comparing survey-weighted sample means. Higher estimates of sensitive behaviours or experiences are assumed to be more accurate.^46^

As a secondary analysis, we also estimated the prevalence of contact IPV (any physical or sexual IPV), overall and by background characteristics, for both the respondent sample and the adjusted confidante sample. We tested for significant differences in contact IPV between respondents and confidantes using z-tests of survey-weighted means.

All analyses were conducted in Stata V.19.5 and include survey weights to account for the complex survey design, including probability of selection and nonresponse. We determined significance using a cut-off of p<0.05.

### Ethical protections

Prior to data collection, interviewers were trained in privacy and confidentiality to ensure participant safety during and after the interview. All respondents aged 18–49 provided verbal informed consent to participate in the survey. In Burkina Faso, girls aged 15–17 provided verbal assent and a parent or guardian provided verbal consent, while in the DRC, girls aged 15–17 were treated as adults and provided verbal consent, consistent with local institutional review board approval. Consent was documented by trained interviewers using the ODK software. Prior to beginning the GBV section of the survey specifically, participants in both countries provided additional consent, and the interviewers re-confirmed full privacy. Referral services were offered to all women regardless of inclusion in the GBV module or disclosure of a violence experience. All procedures are in line with the WHO’s Researching Violence against Women protocols.^3^

### Patient and public involvement

The GBV module was included within select PMA surveys at the request of the Ministries of Health. Results are disseminated to in-country stakeholders at national and local events.

## Results

Analytical samples included a subset of respondents and their confidantes. Respondents were not administered the GBV module if there were privacy concerns (n=60 Burkina Faso; n=8 Kinshasa, DRC; n=82 Kongo Central, DRC). IPV items were only administered to partnered women. Final respondent samples included 3047 women in Burkina Faso, 702 women in Kinshasa, DRC and 688 women in Kongo Central, DRC. Unadjusted confidante samples were also limited to partnered women; final unadjusted confidante samples included 2064 women in Burkina Faso, 304 women in Kinshasa, DRC and 393 women in Kongo Central, DRC, as not all respondents reported a confidante or a confidante that was partnered. The adjusted confidante samples included all reported confidantes as well as ‘missing’ confidantes (ie, for respondents who reported no partnered confidante), thus the final adjusted confidante samples mirrored the overall respondent samples in each site (n=3047 in Burkina Faso, n=702 in Kinshasa, n=688 in Kongo Central), inclusive of 983 ‘missing’ confidantes in Burkina Faso (32.3% of total adjusted sample), 398 in Kinshasa (56.7%) and 295 in Kongo Central (42.9%).

### Assumption 1: confidante sample representativeness

Table 1 shows the demographic characteristics of the respondents and those of the unadjusted and adjusted confidante samples. In Kinshasa, most respondents were aged 30–39 (40.8%), had secondary level education (70.9%) and had three or more children (54.7%); the characteristics of their unadjusted and adjusted partnered confidantes were similar. In Kongo Central, most respondents were 15–29 (42.0%), had secondary level education (55.9%) and had three or more children (66.9%). Unadjusted and adjusted confidantes had comparable education and parity to respondents; however, unadjusted confidantes were slightly older than respondents, whereas adjusted confidantes were similar in age to respondents. While all respondents lived in rural areas, some of their unadjusted and adjusted confidantes lived in urban localities (7.8% and 4.4%, respectively). In Burkina Faso, the majority of respondents were aged 20–29 (42.0%), had no formal education (67.0%), lived in rural areas (80.4%) and had three or more children (62.2%); while age and education were similar across samples (respondent, unadjusted and adjusted confidante), the distribution of the adjusted confidante sample significantly differed from the respondent sample by residence and parity (p<0.05), though the absolute differences were small at ±2 percentage points.

**Table 1:** Characteristics of partnered female respondents aged 15–49 and their closest partnered female confidantes aged 15–49 in the DRC and Burkina Faso*

	DRC					
	Kinshasa			Kongo Central		
	Respondent (N=702)	Unadjusted confidante (N=304)	Adjusted confidante† (N=702)	Respondent (N=688)	Unadjusted confidante (N=393)	Adjusted confidante† (N=699)
	% (n)	% (n)	% (n)	% (n)	% (n)	% (n)
Age						
15–29	33.9 (235)	19.3 (55)	31.9 (206)	42.0 (295)	33.2 (128)	41.2 (271)
30–39	40.8 (301)	46.4 (141)	41.4 (299)	37.6 (254)	40.0 (150)	37.4 (256)
40–49	25.2 (166)	34.3 (101)	26.7 (197)	20.4 (139)	26.8 (96)	21.4 (161)
Education						
None/primary	8.9 (65)	9.2 (30)	8.9 (66)	42.2 (255)	40.9 (141)	42.8 (258)
Secondary	70.9 (504)	66.9 (195)	71.4 (496)	55.9 (411)	56.2 (227)	54.8 (399)
Higher	20.3 (133)	23.9 (74)	19.7 (140)	1.9 (22)	3.0 (20)	2.4 (31)
Wealth tertile						
Poorest	33.1 (257)	−	−	32.1 (167)	−	−
Middle wealth	34.6 (246)	−	−	35.1 (203)	−	−
Wealthiest	32.3 (199)	−	−	32.8 (318)	−	−
Residence						
Rural	−	0.4 (2)	0.2 (2)	100.0 (688)	92.2 (364)	95.6 (659)
Urban	100.0 (702)	99.6 (302)	99.8 (700)	−	7.8 (29)	4.4 (29)
Parity						
0	8.0 (48)	6.5 (19)	7.1 (47)	3.3 (31)	5.7 (22)	5.0 (44)
1–2	37.3 (262)	34.9 (106)	38.0 (262)	29.8 (223)	36.1 (148)	31.3 (249)
3+	54.7 (392)	58.6 (179)	55.0 (393)	66.9 (434)	58.3 (220)	63.7 (395)
Total	100.0 (702)	100.0 (304)	100.0 (702)	100.0 (688)	100.0 (393)	100.0 (688)

	Burkina Faso				
	Respondent (N=3047)	Unadjusted confidante (N=2064)		Adjusted confidante† (N=3047)	
	% (n)	% (n)		% (n)	
Age					
15–29	7.1 (176)	5.7 (89)		6.4 (159)	
20–29	42.0 (1264)	40.8 (717)		43.3 (1149)	
30–39	34.3 (1094)	34.6 (802)		33.4 (1139)	
40–49	16.6 (513)	18.9 (429)		16.9 (600)	
Education					
None	67.0 (1605)	70.5 (1193)		67.6 (1658)	
Primary	18.5 (682)	15.4 (381)		17.3 (608)	
Secondary/higher	14.4 (758)	14.1 (481)		15.1 (779)	
Wealth tertile					
Poorest	35.1 (633)	−		−	
Middle wealth	33.8 (694)	−		−	
Wealthiest	31.1 (1720)	−		−	
Residence					
Rural	80.4 (1371)	80.2 (1001)		**78.5** (**1367**)	
Urban	19.6 (1676)	19.8 (1063)		**21.6** (**1680**)	
Parity					
0	4.6 (182)	5.8 (113)		**6.0 (191**)	
1–2	33.2 (1152)	32.3 (736)		**33.3** (**1146**)	
3+	62.2 (1712)	61.9 (1215)		**60.7** (**1709**)	
Total	100.0 (3047)	100.0 (2064)		100.0 (3047)	

* Estimates weighted, ns unweighted; bold indicates p value<0.05 for design-based F test comparing adjusted confidantes to respondents.

† Estimate includes respondent characteristics in place of ‘missing’ confidantes; post-stratification weights applied.

DRC, Democratic Republic of the Congo.

### Assumption 2: transmission of IPV experiences

Table 2 presents demographic characteristics of respondents overall and by whether they reported having at least one partnered confidante. The proportion of respondents reporting a partnered confidante varied by site (Kinshasa: 43.3%; Kongo Central: 57.1%; Burkina Faso: 67.7%). Women who reported at least one confidante were similar to those who reported no confidantes across age, education and wealth in Kinshasa and Kongo Central; however, women who reported no confidantes in Kongo Central had significantly lower parity than women who reported at least one confidante (parity was not significantly different across groups in Kinshasa). In Burkina Faso, women who reported no confidantes were similar in age to those who reported at least one confidante, but they differed significantly by education, wealth, residence and parity (p<0.05), suggesting that the likelihood of reporting a confidante may vary systematically by socio-demographic characteristics.

**Table 2:** Characteristics of partnered female respondents aged 15–49, overall and by whether they report having at least one close partnered female confidante aged 15–49 in the DRC and Burkina Faso*

	DRC					
	Kinshasa			Kongo Central		
	All respondents (N=702)	0 confidantes (N=398)	≥ 1 confidante (N=304)	All respondents (N=688)	0 confidantes (N=295)	≥ 1 confidante (N=393)
	% (n)	% (n)	% (n)	% (n)	% (n)	% (n)
Age						
15–29	33.9 (235)	38.2 (148)	28.8 (87)	42.0 (295)	47.0 (135)	38.4 (160)
30–39	40.8 (301)	37.1 (155)	45.3 (146)	37.6 (254)	35.6 (102)	39.0 (152)
40–49	25.2 (166)	24.8 (95)	25.8 (71)	20.4 (139)	17.4 (58)	22.6 (81)
Education						
None/primary	8.9 (65)	8.1 (34)	9.7 (31)	42.2 (255)	46.3 (115)	39.1 (140)
Secondary	70.9 (504)	74.4 (298)	66.6 (206)	55.9 (411)	51.0 (169)	59.5 (242)
Higher	20.3 (133)	17.5 (66)	23.6 (67)	1.9 (22)	2.7 (11)	1.4 (11)
Wealth tertile						
Poorest	33.1 (257)	34.1 (147)	32.0 (110)	32.1 (167)	33.4 (71)	31.1 (96)
Middle wealth	34.6 (246)	37.2 (148)	31.6 (98)	35.1 (203)	33.5 (83)	36.3 (120)
Wealthiest	32.3 (199)	28.8 (103)	36.4 (96)	32.8 (318)	33.1 (141)	32.5 (177)
Residence						
Rural	−	−	−	100.0 (688)	100.0 (295)	100.0 (393)
Urban	100.0 (702)	100.0 (398)	100.0 (304)	−	−	−
Parity						
0	8.0 (48)	7.8 (28)	8.3 (20)	3.3 (31)	5.9 (21)	**1.4 (10**)
1–2	37.3 (262)	40.0 (156)	34.1 (106)	29.8 (223)	29.9 (100)	**29.7 (123**)
3+	54.7 (392)	52.3 (214)	57.6 (178)	66.9 (434)	64.2 (174)	**68.8 (260**)
Total	100.0 (702)	100.0 (398)	100.0 (304)	100.0 (688)	100.0 (295)	100.0 (393)

	Burkina Faso					
	All respondents (N=3047)		0 confidantes (N=983)		≥1 confidante (N=2064)	
	% (n)		% (n)		% (n)	
Age						
15–29	7.1 (176)		7.4 (70)		7.0 (106)	
20–29	42.0 (1264)		42.9 (421)		41.7 (843)	
30–39	34.3 (1094)		32.9 (324)		34.8 (770)	
40–49	16.6 (513)		16.8 (168)		16.5 (345)	
Education						
None	67.0 (1605)		63.0 (461)		**68.5** (**1144**)	
Primary	18.5 (682)		18.4 (223)		**18.6 (459**)	
Secondary/higher	14.4 (758)		18.5 (297)		**12.9 (461**)	
Wealth tertile						
Poorest	35.1 (633)		27.8 (149)		**37.8 (484**)	
Middle wealth	33.8 (694)		32.1 (196)		**34.5 (498**)	
Wealthiest	31.1 (1720)		40.0 (638)		**27.7** (**1082**)	
Residence						
Rural	80.4 (1371)		73.2 (366)		**83.1** (**1005**)	
Urban	19.6 (1676)		26.8 (617)		**16.9** (**1059**)	
Parity						
0	4.6 (182)		6.6 (78)		**3.8 (104**)	
1–2	33.2 (1152)		35.9 (410)		**32.2 (742**)	
3+	62.2 (1712)		57.5 (494)		**64.0** (**1218**)	
Total	100.0 (3047)		100.0 (983)		100.0 (2064)	

* Estimates weighted, ns unweighted; bold indicates p value <0.05 for design-based F test comparing respondents with 0 to ≥1 confidante.

DRC, Democratic Republic of the Congo.

Among respondents who reported having a confidante and experiencing IPV, 94.2% in Kinshasa and 87.0% in Kongo Central directly told their confidante about their IPV experience. The corresponding transmission bias adjustment factors were 1.06 (ie, 1/.94) and 1.15 (ie, 1/.87) in Kinshasa and Kongo Central, respectively.

### Assumption 3: reduced social desirability bias when reporting on confidante’s IPV experiences as opposed to reporting own experiences

The prevalence of past-year IPV varied between respondents and confidantes across sites and after adjustments given assumption violations (table 3). In Kinshasa, 35.3% of respondents experienced any IPV compared with 33.0% of unadjusted confidantes. Within the partially adjusted confidante sample, which includes confidantes in place of ‘missing’ confidantes, 33.9% experienced any IPV. Within the fully adjusted confidante sample, which accounts for transmission bias between respondents and confidantes, 36.1% experienced any IPV, which was not significantly different from the respondent estimate of 35.3%. In Kongo Central, the prevalence of any IPV was 29.7% among respondents, 32.7% among unadjusted confidantes, 33.9% among partially adjusted confidantes and 39.0% among fully adjusted confidantes. The prevalence of any IPV among respondents and the fully adjusted sample of confidantes were not significantly different. In Burkina Faso, the prevalence of any IPV was 25.7% among respondents, 24.1% among unadjusted confidantes, and 26.0% among partially adjusted confidantes (adjustment for transmission bias was not possible). Any IPV among respondents and the partially adjusted sample of confidantes was not significantly different.

**Table 3:** Prevalence of IPV among partnered female respondents aged 15–49 and their partnered female confidantes aged 15–49 in the DRC and Burkina Faso*

	DRC							
	Kinshasa				Kongo Central			
	Respondent (N=702)	Unadjusted confidante (N=304)	Partially adjusted confidante† (N=702)	Fully adjusted confidante‡ (N=702)	Respondent (N=688)	Unadjusted confidante (N=393)	Partially adjusted confidante† (N=688)	Fully adjusted confidante‡ (N=688)
	% (95% CI) (n)	% (95% CI) (n)	% (95% CI)	% (95% CI)	% (95% CI) (n)	% (95% CI) (n)	% (95% CI)	% (95% CI)
Emotional IPV	27.6 (22.0 to 34.0) (188)	27.0 (21.3 to 33.7) (82)	27.7 (26.4 to 30.9)	--	24.0 (16.8 to 33.1) (170)	26.2 (17.8 to 36.8) (104)	27.0 (21.5 to 32.4)	--
Physical IPV	12.5 (9.1 to 16.9) (87)	14.9 (10.2 to 21.2) (50)	15.9 (12.7 to 19.0)	--	11.9 (7.5 to 18.4) (95)	16.7 (10.8 to 24.9) (67)	17.7 (13.3 to 22.1)	--
Sexual IPV	12.1 (8.0 to 18.0) (95)	10.0 (6.5 to 15.2) (31)	10.5 (8.6 to 12.4)	--	12.2 (6.8 to 21.0) (88)	12.5 (6.1 to 23.9) (48)	13.5 (8.5 to 18.5)	--
Contact IPV	20.6 (15.0 to 27.5) (148)	19.9 (14.5 to 26.6) (64)	21.1 (17.7 to 24.6)	--	18.0 (11.7 to 26.7) (139)	23.0 (14.4 to 34.7) (90)	24.4 (18.5 to 30.2)	--
Any IPV	35.3 (28.2 to 43.2) (246)	33.0 (26.0 to 40.7) (101)	33.9 (29.9 to 37.9)	36.1 (31.8 to 40.3)	29.7 (21.1 to 40.0) (212)	32.7 (22.3 to 45.1) (130)	33.9 (27.5 to 40.4)	39.0 (31.6 to 46.5)

	Burkina Faso			
	Respondent (N**=3047**)	Unadjusted confidante (N**=2064**)	Partially adjusted confidante**†** (N**=3047**)	Fully adjusted confidante**‡** (N**=3047**)
	% (**95% CI**) **(n)**	% (**95% CI**) **(n)**	% (**95% CI**)	% (**95% CI**)
Emotional IPV	22.9 (19.5 to 26.8) (842)	22.6 (18.6 to 27.2) (583)	24.5 (21.2 to 27.7)	--
Physical IPV	4.5 (3.6 to 5.7) (158)	7.5 (5.6 to 10.1) (164)	**7.8 (6.2 to 9.4**)	--
Sexual IPV	6.4 (4.7 to 8.7) (175)	5.3 (3.8 to 7.2) (104)	5.4 (4.2 to 6.5)	--
Contact IPV	9.4 (7.5 to 11.6) (279)	10.6 (8.2 to 13.7) (221)	10.9 (8.9 to 12.9)	--
Any IPV	25.7 (22.2 to 29.5) (904)	24.1 (19.8 to 29.0) (608)	26.0 (22.6 to 29.4)	--

* Estimates weighted, ns unweighted; bold indicates p value<0.05 between adjusted confidante and respondent samples based on z-tests of survey-weighted means.

† Estimate includes respondent characteristics in place of ‘missing’ confidantes; post-stratification weights applied.

‡ Estimate adjusted for transmission bias, only possible in DRC sites and only possible for any IPV overall; ns not reported for partially and fully adjusted confidante estimates, which represent model-predicted mean proportions rather than direct sample observations.

DRC, Democratic Republic of the Congo; IPV, intimate partner violence.

Past-year emotional IPV was the most prevalent form of IPV experienced among both respondents and confidantes across sites, with slightly lower prevalence among respondents when compared with adjusted confidantes across sites (27.6% vs 27.7% in Kinshasa; 24.0% vs 27.0% in Kongo Central; 22.9% vs 24.5% in Burkina Faso), though differences were not statistically significant. Past-year contact IPV (physical and/or sexual) was slightly higher among adjusted confidantes when compared with respondents across sites—21.1% versus 20.6% in Kinshasa, 24.4% versus 18.0% in Kongo Central and 10.9% versus 9.4% in Burkina Faso—but differences were not statistically significant. When assessing contact IPV by subtype, physical IPV was higher among adjusted confidantes when compared with respondents across sites (15.9% confidantes vs 12.5% respondents in Kinshasa; 17.7% confidantes vs 11.9% respondents in Kongo Central; 7.8% confidantes vs 4.5% respondents in Burkina Faso, p<0.05). However, sexual IPV was higher among respondents when compared with adjusted confidantes in Kinshasa and Burkina Faso (12.1% respondents vs 10.5% confidantes in Kinshasa; 6.4% respondents vs 5.4% confidantes in Burkina Faso), though not statistically significantly so; it was slightly higher among confidantes when compared with respondents in Kongo Central (13.5% vs 12.2%), but similarly, estimates were not statistically significantly different.

Table 4 presents the prevalence of contact IPV by socio-demographic subgroups for both respondents and adjusted confidantes. In Burkina Faso, the prevalence of contact IPV significantly differed between respondents and confidantes by age group, with significantly higher proportions of confidantes aged 40+ reporting contact IPV than respondents of the same age group (11.4% vs 5.2% for confidantes and respondents, respectively). No other significant differences in contact IPV were detected between respondents and confidantes across socio-demographic subgroups. However, some subgroups had small sample sizes, as evidenced by wide CIs, which may have limited power to detect differences.

**Table 4:** Prevalence of contact IPV among partnered female respondents aged 15–49 and their partnered female confidantes aged 15–49 in the DRC and Burkina Faso, by background characteristics*

	Kinshasa		Kongo central	
	Respondent	Adjusted confidante†	Respondent	Adjusted confidante†
	Row % (95% CI) (N subgroup)	Row % (95% CI)	Row % (95% CI) (N subgroup)	Row % (95% CI)
Age				
15–29	24.8 (16.8 to 35.0)(235)	26.3 (21.5 to 31.3)	20.5 (13.0 to 30.7)(295)	23.2 (17.7 to 28.7)
30–39	18.6 (12.5 to 26.8)(301)	19.2 (14.9 to 23.5)	17.3 (10.0 to 28.3)(254)	26.9 (18.9 to 34.9)
40–49	18.1 (10.1 to 30.2) (166)	18.0 (12.5 to 22.5)	14.2 (7.8 to 24.6) (139)	22.3 (13.0 to 31.5)
Education				
Never/primary	24.3 (12.3 to 42.1) (65)	28.1 (18.1 to 38.1)	19.8 (10.4 to 34.4) (255)	27.6 (19.6 to 35.6)
Secondary	23.6 (17.3 to 31.3) (504)	22.2 (18.6 to 25.8)	16.9 (11.3 to 24.5) (411)	22.3 (16.0 to 28.6)
Higher	8.7 (3.3 to 20.6) (133)	14.0 (9.0 to 19.1)	9.6 (1.6 to 41.2) (22)	12.9 (5.4 to 20.4)
Wealth tertile				
Poorest	32.3 (23.0 to 43.1) (257)	−	17.2 (6.5 to 38.0) (167)	−
Middle wealth	12 (7.4 to 19.0) (246)	−	24.1 (15.1 to 36.1) (203)	−
Wealthiest	17.8 (11.1 to 27.3) (199) to	−	12.3 (8.5 to 17.4) (318)	−
Residence				
Rural	−	0 (–)	18.0 (11.7 to 26.7) (688)	23.6 (17.7 to 29.5)
Urban	20.6 (15.0 to 27.5) (702)	21.2 (17.7 to 24.6)	−	40.8 (20.7 to 61.0)
Parity				
0	16.3 (6.8 to 34.1) (48)	25.2 (16.1 to 34.3)	14.4 (4.0 to 40.5) (31)	27.7 (18.4 to 37.1)
1–2	21.0 (14.7 to 29.2) (262)	20.5 (16.7 to 24.4)	18.4 (11.7 to 27.6) (223)	17.0 (10.8 to 23.2)
3+	20.9 (15.3 to 27.9) (392)	21.0 (16.5 to 25.6)	18.0 (11.3 to 27.5) (434)	27.7 (21.2 to 34.3)
Total	20.6 (15.02 to 7.5) (702)	21.1 (17.7 to 24.6)	18.0 (11.7 to 26.7) (688)	24.4 (18.5 to 30.2)

	Burkina Faso			
	Respondent		Adjusted confidante**†**	
	Row % (**95% CI**) **(N subgroup)**		Row % (**95% CI**)	
Age				
15–19	4.2 (1.8 to 9.2) (176)		10.0 (4.8 to 15.3)	
20–29	10.8 (7.9 to 14.7) (1264)		10.4 (8.0 to 12.8)	
30–39	10.6 (8.0 to 13.9) (1094)		11.4 (9.0 to 13.8)	
40–49	**5.2 (3.2 to 8.6) (513**)		**11.4 (8.3 to 14.6**)	
Education				
Never	9.2 (7.0 to 11.9) (1605)		10.6 (8.2 to 13.0)	
Primary	9.0 (6.1 to 13.0) (682)		11.1 (8.4 to 13.8)	
Secondary/higher	10.7 (7.1 to 15.9) (758)		12.2 (8.9 to 15.5)	
Wealth tertile				
Poorest	9.0 (6.5 to 12.2) (633)		−	
Middle wealth	11.8 (8.6 to 15.9) (694)		−	
Wealthiest	7.1 (5.4 to 9.4) (1720)		−	
Residence				
Rural	9.9 (7.6 to 12.7) (1371)		11.3 (8.8 to 13.7)	
Urban	7.3 (5.5 to 9.7) (1676)		9.6 (8.1 to 11.1)	
Parity				
0	5.4 (2.0 to 13.8) (182)		11.0 (6.0 to 16.1)	
1–2	8.7 (6.6 to 11.3) (1152)		9.3 (7.2 to 11.4)	
3+	10.0 (7.7 to 12.9) (1712)		11.8 (9.4 to 14.1)	
Total	9.5 (7.5 to 11.6) (3047)		10.9 (8.9 to 12.9)	

* Estimates weighted, ns unweighted; bold indicates p value<0.05 between adjusted confidante and respondent samples based on z-tests of survey-weighted means.

† Estimates include respondent characteristics in place of ‘missing’ confidantes; post-stratification weights applied; ns not reported for confidante estimates, which represent model-predicted mean proportions rather than direct sample observations.

DRC, Democratic Republic of the Congo; IPV, intimate partner violence.

## Discussion

To our knowledge, this is the first study to apply the confidante methodology to IPV measurement. Overall, the confidante method produced comparable estimates to respondents’ direct reports across sites (35.3% respondent vs 36.1% fully adjusted confidante in Kinshasa; 29.7% respondent vs 39.0% fully adjusted confidante in Kongo Central; 25.7% respondent vs 26.0% partially adjusted confidante in Burkina Faso, not statistically significantly different). Though we have no way to validate our estimates given the lack of external, objective measures, our overall IPV estimates, both direct and confidante derived, are comparable to recent national estimates from Burkina Faso and the DRC using direct survey questions (22% and 36%, respectively),^47 48^ indicating that population-based surveys with direct IPV questions are reliable when implemented under recommended ethical guidelines.

Despite comparable estimates in overall IPV prevalence between respondents and adjusted confidantes, there were notable differences in patterns by IPV subtype. Across sites, physical IPV was modestly, consistently lower among respondents (12.5% respondents vs 15.9% adjusted confidantes in Kinshasa; 11.9% respondents vs 17.7% adjusted confidantes in Kongo Central; 4.5% respondents vs 7.8% adjusted confidantes in Burkina Faso), with a statistically significant difference only in Burkina Faso. Previous studies have found that physical injuries and the severity and frequency of IPV are associated with disclosure to informal supports, such as friends and family.^49^,^53^ While we were unable to assess injuries, severity or frequency of physical IPV in our study, these previous findings may explain the increased prevalence of physical IPV we see among adjusted confidante samples, as more severe or recurrent physical violence may have been more likely to be shared. Though physical IPV was lower within the respondent sample, it was not significantly so in the DRC, indicating direct physical IPV estimates may be reliable. Further, a recent study in Tanzania comparing the list experiment to direct reports found comparable estimates across measurement methods and similarly concluded the direct reports to likely be reliable.^10^

In contrast, sexual IPV was higher within the respondent sample as compared with the adjusted confidante sample in Kinshasa and Burkina Faso (12.1% respondents vs 10.5% adjusted confidantes in Kinshasa; 6.4% respondents vs 5.4% adjusted confidantes in Burkina Faso), though these differences were not statistically significant. This finding aligns with previous studies exploring disclosure of sexual IPV. In Norway, women who experienced sexual IPV were less likely to disclose to informal supports, such as friends or family, when compared with those who had experienced physical or emotional IPV.^54^ In a US-based study, women who experienced sexual abuse had decreased odds of seeking help from family compared with those who experienced concurrent physical and sexual abuse.^52^ This potentially indicates that sexual IPV alone is more sensitive than other or concurrent forms of IPV, and women are less likely to disclose experiences of sexual IPV to those in their social network (ie, respondents are less likely to know about their confidante’s sexual IPV). In a Tanzania-based study, the prevalence of sexual IPV was higher using the list experiment method compared with the direct method,^10^ highlighting that direct reports of sexual IPV may still be under-reported. Thus, not only are direct reports likely underestimates, but the confidante method does not appear to produce more accurate results given the sensitivity of sexual IPV and the increased likelihood of non-disclosure. IPV type-specific transmission bias adjustments may improve confidante estimates in future work.

We also found that the prevalence of contact IPV (any physical or sexual IPV) was significantly different by age group between respondents and adjusted confidantes in Burkina Faso, with higher reports of contact IPV among confidantes aged 40+ compared with to respondents of the same age group. This may suggest that transmission of IPV experiences within social networks may become more normalised or women may feel more comfortable sharing as they age, as the length of their friendships (with a confidante) and partnerships are likely longer. This is consistent with results from a study in the USA that found older women were more likely to disclose IPV to family than younger women.^55^

Our study is not without limitations, mainly related to method assumption violations and adjustments. The confidante approach’s use of a substitute confidante for respondents who lack one is meant to ensure that women without confidantes—who we show are different from those who have one—are represented in the surrogate confidante sample, reducing selection bias. For the confidante method to produce unbiased population-level estimates, the surrogate confidante sample must reflect the full population of respondents, including those without confidantes (assumption 1). This is of particular concern given IPV’s links to social isolation and partner control, and could introduce ‘barrier effects’:^17^ if respondents who report confidantes have lower IPV rates than those who do not, their confidantes may also have lower IPV rates, potentially underestimating IPV prevalence in the confidante sample if not adjusted. The consistency of findings across adjusted and unadjusted samples provides partial reassurance this bias may not be substantial. Second, transmission of IPV experiences between respondents and confidantes was incomplete in both DRC sites (violation of assumption 2), and we had no information about the extent of transmission in Burkina Faso. While we were able to adjust for transmission bias in Kinshasa and Kongo Central, we were unable to do so in Burkina Faso, preventing us from generating a fully adjusted IPV estimate among confidantes in that setting. Given that the transmission bias factor did not lead to significant differences in estimates in the DRC sites and patterns were largely consistent, inclusion of this adjustment factor would likely not change our conclusions in Burkina Faso, unless IPV disclosure is lower (and thus the adjustment for transmission bias higher) in this context.

A few other limitations should be considered when assessing findings. While we identified whether or not respondents had confidantes (‘someone with whom you share very personal information’), we were not able to distinguish the type of confidante (family vs friend). Previous research has indicated that the likelihood of disclosure of sexual IPV is dependent on the type of relationship: individuals experiencing sexual abuse in isolation are less likely to seek help from family but not from friends, as compared with people experiencing concurrent physical and sexual abuse.^52^ Sexual IPV may be misreported within the confidante sample if transmission of IPV experiences is dependent on the type of confidante, which can then lead to underestimation of our overall IPV estimate (given some people experience sexual IPV in isolation and would not be captured via other IPV types). Some respondents could have confidantes that were not captured given our sample criteria (ie, partnered, aged 15–49, living in the same country), which could have excluded meaningful confidante relationships in this analysis. However, including them could introduce selection bias by reducing comparability between the respondent and confidante samples, a core requirement of assumption 1. When adjusting for transmission of IPV experiences, we assumed equal sharing between respondents and confidantes, as has been described in previous applications of the confidante method; however, this may not be the case. A study in Ethiopia found that those who experienced only one type of IPV were less likely to disclose their experiences compared with those who experienced multiple types of violence.^56^ Further, injuries and violence severity and frequency have been found to be associated with disclosure to informal supports.^49^,^53^ As such, differing experiences of severity, frequency and concurrent violence may not yield equal sharing of experiences between individuals, rendering our transmission bias adjustment factor inadequate for capturing this nuance in differential disclosure.

There may also be a risk of multiple respondents reporting on the same confidante, as some women are more popular than others. However, we believe this is unlikely to occur (or at least not frequently enough to bias our estimates) given our multistage stratified cluster sampling design at the national (Burkina Faso) and subnational (DRC) levels. The geographical distribution and random selection processes—including random selection of enumeration areas, households within areas and one woman per household for the GBV module, including limiting confidantes to those that are partnered—make it statistically improbable that multiple respondents would identify the same individual as their confidante. Even if the unlikely scenario occurred where a confidante was reported by multiple respondents, the impact on prevalence estimates would likely be minimal. We do acknowledge, however, that differential knowledge about the same person’s experiences across respondents could introduce bias. Similarly, women’s differential probability of selection as someone’s confidante could introduce bias if it systematically differed by whether they have experienced IPV, though we are not able to assess this with our current data. Lastly, the role of one’s confidante in navigating an experience of IPV is less clear, which may have implications for IPV disclosure and the value of this measurement method. The confidante method, which has most frequently been used to estimate abortion incidence, may be more meaningful in measuring behaviours that are legally restrictive and may necessitate disclosure, as is often the case for abortion, where confidantes provide instrumental support in accessing abortion care (ie, providing financial support, recommending abortion method or sources of care, travelling to clinic/procedure with friend).^57 58^ These support-based dynamics may be different for those experiencing IPV.

Despite these limitations, our study has several strengths. To our knowledge, this is the first application of the confidante method for IPV estimation. While the confidante method has a number of limitations, implementing the method for IPV estimation and assessing its performance contributes to the growing body of literature on indirect estimation techniques for various sensitive topics. These data are from nationally or subnationally representative samples which increase the external validity of our findings, particularly for the directly reported IPV estimates among our respondent samples. We also collected socio-demographic characteristics for both respondents and confidantes, allowing us to analyse IPV by individual-level characteristics, which has not been possible in most other studies applying indirect measurement techniques (ie, the list experiment) to IPV estimation.^11^ Additionally, despite a lack of statistically significant differences for overall IPV between respondents and confidantes, we see suggestive patterns of differential IPV disclosure by IPV subtype.

Overall, the confidante method produced comparable estimates to respondents’ direct reports across sites, indicating that the confidante method is not more advantageous than direct assessment of IPV via self-report on surveys, particularly given violations in confidante method assumptions. As the first study to apply the confidante method to IPV measurement, however, these findings, including null results, represent a meaningful contribution. Establishing what this methodology does and does not offer provides critical groundwork for future researchers. The confidante method may have greater utility for behaviours where disclosure to social networks is more instrumental—such as abortion, where confidantes often play an active role in accessing care—than for IPV, where disclosure is more variable and shaped by sensitivity, shame and partner control. Future work should explore whether refinements such as IPV subtype-specific transmission bias adjustments could improve its performance. In the meantime, the WHO’s Researching Violence Against Women and the United Nation’*s* Guidelines for Producing Statistics on Violence against Women highlight the quality of population-based survey data when appropriate ethical protections are in place, minimising the potential for IPV under-reporting.^3 4^ Data for this study were collected in accordance with global guidelines, ensuring ethical protections were in place at every stage of the data collection process (ie, training interviewers to handle sensitive survey topics, sex-matching respondents and interviewers, only allowing one woman per household to participate in the GBV module, programming privacy checks into the data collection software, stopping the interview if privacy is compromised). Moreover, training emphasises minimising shame and blame, empathetic listening, support and referral to accessible GBV response services. These protocols may contribute to easing respondent disclosure and producing more accurate prevalence estimates using direct reporting. High quality surveillance is needed to continue to monitor IPV, which is a threat to women’s health and well-being.

## Data Availability

Data are available in a public, open access repository.
