# Supplementary material for: Measurement innovation for sensitive behaviours: applying direct and social network-based estimation approaches to intimate partner violence in Burkina Faso and the Democratic Republic of the Congo using cross-sectional data
Source: BMJ Open. 2026 May 26;16(5):e114411. doi: 10.1136/bmjopen-2025-114411 (PMC13218162; doi:10.1136/bmjopen-2025-114411)
Supplement: Supplementary file 1 [file bmjopen-2025-114411.reviewer_comments.pdf]

PEER REVIEW HISTORY

BMJ Open publishes all reviews undertaken for accepted manuscripts. Reviewers are asked to complete a checklist review form and are provided with free text boxes to elaborate on their assessment. These free text comments are reproduced below.

ARTICLE DETAILS

Title (Provisional)

Measurement innovation for sensitive behaviors: Applying direct and social network-based estimation approaches to intimate partner violence in Burkina Faso and the Democratic Republic of the Congo using cross-sectional data

Authors

Thomas, Haley L.; Bell, Suzanne O.; Bazié, Fiacre; Guiella, Georges; Kayembe, Dynah M.; Akilimali, Pierre Z; Decker, Michele; Wood, Shannon

---

VERSION 1 - REVIEW

**Reviewer**	**1**
**Name**	**Shrestha, Prabin**
**Affiliation**	**Tribhuvan University - Tri-Chandra Multiple Campus, Department of Psychology**
**Date**	**03-Feb-2026**
**COI**	**None**

This study explores the feasibility of the confidante methodology to measure past-year intimate partner violence in Burkina Faso and Democratic Republic of Congo by comparing the direct assessment and indirect reporting method using the confidante methods as well as to assess the performance of each confidante method assumption.

The abstract is well discussed.

The introduction section provides sufficient background information about IPV in the two settings as well as the confidante method.

The methods part is fine.

As the study focuses on using and assessing performance of confidante method, there is a need to review by a specialist statistician regarding the methods and the analysis made.

There is a need to clarify the significance of the study as well as the conclusions in terms of the knowledge and evidences it generates in the current body of knowledge given that this was the first study to apply confidante methods to IPV measurement, there was no any

advantage of the confidante methodology over the standard method and in the context of the limitations discussed.

Expert statistical analysis is required.

**Reviewer**	**2**
**Name**	**Ali, Tazeen**
**Affiliation**	**The Aga Khan University Hospital Karachi, School of Nursing and Midwifery & Community Health Sciences Aga Khan University**
**Date**	**05-Feb-2026**
**COI**	**None**

This manuscript addresses IPV with clarity, methodological rigor, and strong ethical grounding. The research question is well-defined, and the analysis is appropriate (although it requires specialist statistical review). The findings contribute meaningfully to the existing literature.

**Reviewer**	**3**
**Name**	**Asghar, Khudejha**
**Affiliation**	**The University of Edinburgh School of Social and Political Science, Social Work**
**Date**	**16-Feb-2026**
**COI**	**None**

Lines 485-487, "Previous research has indicated that the likelihood of disclosure of sexual IPV…" – the wording in latter half of this sentence is confusing, please clarify if people are more likely to seek support from family or friends for sexual IPV.

**Reviewer**	**4**
**Name**	**Dack, Kyle**
**Affiliation**	**University of Bristol, MRC Integrative Epidemiology Unit**
**Date**	**08-Mar-2026**
**COI**	**None**

Statistical review summary:

Overall, the paper is very well written. The design and package of analyses is novel, and it was interesting to read. My only major query relates to how the study handles respondents

who reported not having a confidante. This is mostly treated as a missing data issue, with a form of imputation used to replace the missing data with surrogate confidantes.

This affected a notable proportion of the study participants (approximately 30%), and changes the comparisons substantially (e.g. Table 1 unadjusted vs adjusted confidantes). As such, it is important to communicate and evaluate the missing data assumptions - e.g. describe the pattern of missingness, and evaluate what it represents (missing confidante or true absence of a confidante).

Abstract:

1. The abstract contains all key methodological details, the results are clear - no suggestions.

Introduction:

2. The introduction aligns well with STROBE guidelines and was interesting to read.

Methods:

3. Overall the methodology is strong - the procedure and rationale are very clear, and the statistical analysis looks reasonable. However, I have several queries, mostly related to the confidante data and handling of missing data:

4. Respondents were asked if they had a confidante between the ages of 15-49. Is there a rationale for this age range? I see that the survey included only women in this age range, but couldn't a confidante be older?

In "measures" it is reported that a complete-case approach was used. However, assumption 1 involves treating a lack of a confidante as missing data, and imputing details based on the respondent's characteristics. I am unsure how this remains a complete-case analysis.

5. Confidante surrogates: Line 248-249 contains an assumption that if a respondent reported having no surrogate, this reflects missing information. This is possible, but it is also possible that an individual may truly have no surrogate. Particularly given that IPV has links to social isolation, partner control, etc, and there could be an association between the degree of IPV and the presence or absence of a confidante.

-I am not an expert in IPV but suggest this assumption could be made more explicit and given a stronger justification, as it is currently unstated and it affects approximately 33% of the sample. (it is not reported but I think 983 of the 3047 respondents were assigned a surrogate). There is already information in the results which can be used to evaluate the assumption, e.g. table 2, table 3, which contain the "sensitivity analyses" I would otherwise have suggested - comparing those with/without a surrogate, and complete case analyses (unadjusted confidante). But I suggest that this could be evaluated more directly in the discussion, as it seems a key assumption.

6. Line 260: Which form of regression was used, including which covariates?

Results:

7. I suggest commenting on the number of respondents who did not report a confidante and were given a surrogate, and to briefly comment on pattern of this missingness / lack of a confidante. I believe the total number is 983, but table 1 indicates there is notable variation, e.g. 56% in Kinshasa and 33% in Burkina Faso. There is also variation by characteristic which indicates the lack of a confidante is not completely random.

8. Overall the in-text results and tables are clear - I did not find anything inconsistent, unclear, and I have no additional queries. Similarly, the discussion is well written and supported by the findings of the study. My only suggestion is in one or both of these sections to discuss more the issue of those with no confidante. Line 483 for example seems to interpret that those who reported no confidante truly did not have one. Wouldn't this make it unsuitable to then assign these respondents a surrogate confidante?

VERSION 1 - AUTHOR RESPONSE

Reviewer: 1
Mr. Prabin Shrestha, Tribhuvan University - Tri-Chandra Multiple Campus
Comments to the Author:

This study explores the feasibility of the confidante methodology to measure past-year intimate partner violence in Burkina Faso and Democratic Republic of Congo by comparing the direct assessment and indirect reporting method using the confidante methods as well as to assess the performance of each confidante method assumption. The abstract is well discussed. The introduction section provides sufficient background information about IPV in the two settings as well as the confidante method. The methods part is fine. As the study focuses on using and assessing performance of confidante method, there is a need to review by a specialist statistician regarding the methods and the analysis made.

> **<u>Author Response:</u> We thank the reviewer for their positive assessment of the abstract, introduction, and methods sections.**

There is a need to clarify the significance of the study as well as the conclusions in terms of the knowledge and evidences it generates in the current body of knowledge given that this was the first study to apply confidante methods to IPV measurement, there was no any advantage of the confidante methodology over the standard method and in the context of the limitations discussed. Expert statistical analysis is required.

> **<u>Author Response:</u> We thank the reviewer for raising these points. We have expanded our conclusions to address them: "As the first study to apply the confidante method to IPV measurement, however, these findings, including null results, represent a meaningful contribution. Establishing what this methodology does and does not offer provides critical groundwork for future researchers seeking to improve survey-based measurement of sensitive topics. The confidante method may have greater utility for behaviors where disclosure to social networks is more instrumental—such as abortion, where confidantes often play an active role in accessing care—than for IPV, where disclosure is more variable and shaped by sensitivity, shame, and partner control.**

Future work should explore whether refinements, such as IPV subtype-specific transmission bias adjustments, could improve its performance." (pgs 20-21, lines 559-566)

Reviewer: 2
Dr. Tazeen Ali, The Aga Khan University Hospital Karachi
Comments to the Author:

This manuscript addresses IPV with clarity, methodological rigor, and strong ethical grounding. The research question is well-defined, and the analysis is appropriate (although it requires specialist statistical review). The findings contribute meaningfully to the existing literature.

Author Response: We thank the reviewer for their positive assessment of our manuscript.

Reviewer: 3
Dr. Khudejha Asghar, The University of Edinburgh School of Social and Political Science
Comments to the Author:

Lines 485-487, "Previous research has indicated that the likelihood of disclosure of sexual IPV…" – the wording in latter half of this sentence is confusing, please clarify if people are more likely to seek support from family or friends for sexual IPV.

Author Response: Thank you for this comment. We have updated this sentence to improve clarity. It now reads: "Previous research has indicated that the likelihood of disclosure of sexual IPV is dependent on the type of relationship: individuals experiencing sexual abuse in isolation are less likely to seek help from family but not from friends, as compared to people experiencing concurrent physical and sexual abuse (50)." (pg 19, lines 501-504)

Reviewer: 4
Dr. Kyle Dack, University of Bristol
Comments to the Author:

Statistical review summary:
Overall, the paper is very well written. The design and package of analyses is novel, and it was interesting to read. My only major query relates to how the study handles respondents who reported not having a confidante. This is mostly treated as a missing data issue, with a form of imputation used to replace the missing data with surrogate confidantes. This affected a notable proportion of the study participants (approximately 30%), and changes the comparisons substantially (e.g. Table 1 unadjusted vs adjusted confidantes). As such, it is important to communicate and evaluate the missing data assumptions - e.g. describe the pattern of missingness, and evaluate what it represents (missing confidante or true absence of a confidante).

<u>Author Response:</u> Thank you for your thorough review of our manuscript. We address your specific questions below.

The abstract contains all key methodological details, the results are clear - no suggestions. The introduction aligns well with STROBE guidelines and was interesting to read.

<u>Author Response:</u> We thank the reviewer for their positive assessment of the abstract and introduction.

Overall the methodology is strong - the procedure and rationale are very clear, and the statistical analysis looks reasonable. However, I have several queries, mostly related to the confidante data and handling of missing data:

- Respondents were asked if they had a confidante between the ages of 15-49. Is there a rationale for this age range? I see that the survey included only women in this age range, but couldn't a confidante be older?
- In "measures" it is reported that a complete-case approach was used. However, assumption 1 involves treating a lack of a confidante as missing data, and imputing details based on the respondent's characteristics. I am unsure how this remains a complete-case analysis.

<u>Author Response:</u> We thank the reviewer for these questions. A respondent could, in real life, have a confidante outside this age range, but we did not collect data on those confidantes. Our survey was administered to a nationally representative sample of women aged 15-49; the confidante method requires that the confidante sample be representative of the same target population in order to estimate IPV at the national level for that target population using the surrogate confidante data (assumption 1). Including confidantes outside this age range could introduce additional selection bias and reduce comparability between the respondent and confidante samples, a key requirement of assumption 1. We have updated our Methods section to indicate this is based on our survey population: "To identify the confidante sample, respondents were asked if they had a confidante who was a woman between the ages of 15 and 49—consistent with the survey's target population—who lived in the country and with whom they share very personal information." (pg 6, lines 212-214). We acknowledge that this restriction may exclude some meaningful confidante relationships and note it as a limitation of the current approach: "Some respondents could have confidantes that were not captured given our sample criteria (i.e., partnered, aged 15-49, living in the same country), which could have excluded meaningful confidante relationships in this analysis. However, including them could introduce selection bias by reducing comparability between the respondent and confidante samples, a core requirement of assumption 1." (pg 20, lines 507-511).

Thank you for flagging the potential source of confusion regarding complete case analysis. We have updated the sentence to improve clarity: "Respondents missing self-reported IPV data or IPV data for their confidante were excluded from analyses (<1% across sites)." (pg 6, lines 233-235).

Confidante surrogates: Line 248-249 contains an assumption that if a respondent reported having no surrogate, this reflects missing information. This is possible, but it is also possible that an individual may truly have no surrogate. Particularly given that IPV has links to social isolation, partner control, etc, and there could be an association between the degree of IPV and the presence or absence of a confidante. I am not an expert in IPV but suggest this assumption could be made more explicit and given a stronger justification, as it is currently unstated and it affects approximately 33% of the sample. (it is not reported but I think 983 of the 3047 respondents were assigned a surrogate). There is already information in the results which can be used to evaluate the assumption, e.g. table 2, table 3, which contain the "sensitivity analyses" I would otherwise have suggested - comparing those with/without a surrogate, and complete case analyses (unadjusted confidante). But I suggest that this could be evaluated more directly in the discussion, as it seems a key assumption.

Author Response: We thank the reviewer for this observation and agree this warrants more explicit discussion. We acknowledge that both scenarios are plausible—some respondents may genuinely lack a confidante, while others may have such a relationship that was not captured. Importantly, the confidante approach is not simply to create a substitute confidante for women who lack one (or an eligible one), but rather ensures that such women are represented in the confidante sample to reduce selection bias. Given the observed differences between those with and without confidantes, not including women without confidantes in the surrogate confidante sample would bias the sample and associated estimates. We have revised the Discussion to address this more explicitly: "The confidante approach's use of a substitute confidante for respondents who lack one is meant to ensure that women without confidantes—who we show are different from those who have one—are represented in the surrogate confidante sample, reducing selection bias. For the confidante method to produce unbiased population-level estimates, the surrogate confidante sample must reflect the full population of respondents, including those without confidantes. This is of particular concern given IPV's links to social isolation and partner control, and could introduce "barrier effects" (17): if respondents who report confidantes have lower IPV rates than those who do not, their confidantes may also have lower IPV rates, potentially underestimating IPV prevalence in the confidante sample if not adjusted. The consistency of findings across adjusted and unadjusted samples provides partial reassurance this bias may not be substantial." (pg 19, lines 478-488)

Line 260: Which form of regression was used, including which covariates?

Author Response: We have provided additional information to clarify: "We then predicted the probability that the these "missing" confidantes would have experienced past-year IPV by fitting separate logistic regression models for each IPV type, regressing the respondents' sociodemographic characteristics, including age, education, wealth tertile, urban/rural residence and parity, on the corresponding observed confidante IPV data, and imputing the resulting predicted probabilities (ranging from 0 to 1) for these confidantes." (pg 7, lines 261-265) and "For the adjusted confidante sample, prevalence was estimated by taking the mean across

all confidante IPV values, combining binary outcomes (0 or 1) for those with an observed confidante and imputed predicted probabilities (0-1) for "missing" confidantes." (pg 8, lines 289-292)

I suggest commenting on the number of respondents who did not report a confidante and were given a surrogate, and to briefly comment on pattern of this missingness / lack of a confidante. I believe the total number is 983, but table 1 indicates there is notable variation, e.g. 56% in Kinshasa and 33% in Burkina Faso. There is also variation by characteristic which indicates the lack of a confidante is not completely random.

<u>Author Response:</u> We have updated the results section to explicitly report the number and proportion of "missing" confidantes by site, extending the existing sentence on adjusted samples: "The adjusted confidante samples included all reported confidantes as well as "missing" confidantes (i.e., for respondents who reported no partnered confidante), thus the final adjusted confidante samples mirrored the overall respondent samples in each site (n=3,047 in Burkina Faso, n=702 in Kinshasa, n=688 in Kongo Central), inclusive of 983 "missing" confidantes in Burkina Faso (32.3% of total adjusted sample), 398 in Kinshasa (56.7%), and 295 in Kongo Central (42.9%)." (pg 9, lines 336-341) Regarding the pattern of those without a partnered confidante that required a "missing" confidante, Table 2 presents characteristics of respondents by whether they reported 0 versus 1+ partnered confidantes, with slightly updated accompanying text: "In Burkina Faso, women who reported no confidantes were similar in age to those who reported at least one confidante, but they differed significantly by education, wealth, residence, and parity (p<0.05), suggesting that the likelihood of reporting a confidante may vary systematically by sociodemographic characteristics." (pg 11, lines 367-371)

Overall the in-text results and tables are clear - I did not find anything inconsistent, unclear, and I have no additional queries. Similarly, the discussion is well written and supported by the findings of the study. My only suggestion is in one or both of these sections to discuss more the issue of those with no confidante. Line 483 for example seems to interpret that those who reported no confidante truly did not have one. Wouldn't this make it unsuitable to then assign these respondents a surrogate confidante?

<u>Author Response:</u> We thank the reviewer for this observation. As described in our response to the confidante comment above, we have revised the Discussion to explicitly acknowledge that both scenarios are plausible, added language on barrier effects, and note that unadjusted and adjusted confidante estimates are presented throughout (Table 3) so readers can evaluate findings with and without the "missing" confidante adjustment. Additionally, the surrogate approach is not simply to create a substitute confidante for women who lack one, but rather ensures that such women are represented in the confidante sample to reduce selection bias. Given the observed differences between those with and without confidantes, not including these women in the confidante sample would bias the sample and associated estimates.

VERSION 2 - REVIEW

**Reviewer**	**4**
**Name**	**Dack, Kyle**
**Affiliation**	**University of Bristol, MRC Integrative Epidemiology Unit**
**Date**	**01-May-2026**
**COI**	

Thank you to the authors for responding in detail to my comments. I have no further queries - it is a nice paper.
